# Supplementary material for: Contamination, Source Identification, Ecological and Human Health Risks Assessment of Potentially Toxic-Elements in Soils of Typical Rare-Earth Mining Areas
Source: Int J Environ Res Public Health. 2022 Nov 16;19(22):15105. doi: 10.3390/ijerph192215105 (PMC9690513; doi:10.3390/ijerph192215105)
Supplement: Supplementary file 1 [file ijerph-19-15105-s001.zip › ijerph-1995390-supplementary.pdf]

Supplementary Material

Tables

Table.S1 Classification of enrichment factor and nemerow integrated pollution index

Table.S2 Potential ecological risk classification of potentially toxic elements before and after revision.

Table S3 Parameters, input assumptions and calculation formulas for human non-carcinogenic risk assessment

Table S4 Values of RfD (mg/kg/day) for seven potentially toxic elements in the three exposure pathways

Table S5 Average non-carcinogenic exposure of potentially toxic elements in soil under three exposure pathways [mg/(kg·d)]

Table.S6 Evaluation standards of non-carcinogenic risk

Figures

Figure S1 Enrichment index of potentially toxic elements in soil of the study area.

Figure S2 Correlation analysis between potentially toxic elements and physicochemical properties in soil. * represented the significant to probability level 0.05, ** represented the significant to probability level 0.01, and *** represented the significant to probability level 0.001.

Figure S3 Distribution of physicochemical properties in soils in the study area (pH, TC, TN).

Figure S4 Contribution rate of each factor to potentially toxic element concentration sources

Figure S5 The thematic map of the study area. (a) The surface coverage of the study area and the location of the mine sites. (b) The network of roads and waterway and distribution of factory sites around the study area. (c) The subdivision of towns around the study area. (d) Population density in the study area and the distribution of sampling sites.

Table S1. Classification of enrichment factor and nemerow integrated pollution index

Range	Classification	Range	Classification
EF < 2	Depletion to minimum enrichment	NPI < 0.7	Clean
2 ≤ EF< 5	moderate enrichment	0.7 ≤ NPI < 1	Relatively clean
5 ≤EF< 20	significant enrichment	1 ≤ NPI < 2	Lightly contaminated
20 ≤EF< 40	very high enrichment	2 ≤ NPI <3	Contaminated
EF > 40	extremely high enrichment	NPI ≥ 3	Extremely contaminated

$$\mathrm{EF} = \frac{(C_i/C_{ref})_{sample}}{(C_i/C_{ref})_{background}}$$

where C_i is the concentration of the potentially toxic elements (mg/kg); C_{ref} is the concentration of the standardized reference element (mg/kg), and the elements Sc, Mn, Ti, Al and Fe are usually chosen as references. In this study, Sc was used as the reference element because of the relatively high stability of element Sc concentration.

Table S2. Potential ecological risk classification of potentially toxic elements before and after revision.

Classification	E_{ri}		RI	
	Hakanson	This study	Hakanson	This study
Slight	E_{ri} <4 0	E_{ri} < 20	RI < 150	RI < 50
Medium	40 ≤ E_{ri} < 80	20 ≤E_{ri} <40	150 ≤RI< 300	50 ≤ RI < 100
Strong	80 ≤ E_{ri} < 160	40 ≤E_{ri} <80	300 ≤RI< 600	100 ≤RI < 200
Very strong	160 ≤E_{ri}< 320	80 ≤E_{ri}< 160	RI > 600	RI ≥ 400

Table S3. Parameters, input assumptions and calculation formulas for human non-carcinogenic risk assessment

Symbol	Parameter	Unit	Value
C_i	Concentration of an element	mg/kg	
IR_{ing}	Soil ingestion rate	mg/day	200 (for child), 100 (for adult)
EF	Exposure frequency	day/year	350
IR_{inh}	Air inhalation rate	m^3/day	7.5 (for child), 14.5 (for adult)
ED	Exposure duration	year	24 [6 (for child) for non-cancer effects]
SA	Skin area	cm^2	2848.01(for child), 5373.99 (for adult)
SL	Skin adherence factor	mg/cm^2	0.2 (for child), 0.07(for adult)
BW	Body weight	kg	19.2(for child), 61.8(for adult)
AT	Mean Time	day	9125 for non-carcinogens
ABS	Dermal absorption factor	Unitless	0.006(Pb), 0.14(Cd), 0.1(Cu), 0.02(Zn), 0.05(Hg), 0.03(As), 0.001 for rest
PEF	Particle emission factor	m^3/kg	1.36E9
ADD_{ing}	$\frac{C_i \times IR_{ing} \times EF \times ED}{BW \times AT} \times 10^{-6}$		
ADD_{der}	$\frac{C_i \times SA \times SL \times ABS \times EF \times\times ED}{BW \times AT} \times 10^{-6}$		
ADD_{inh}	$\frac{C_i \times IR_{inh} \times EF \times ED}{PEF \times BW \times AT}$		
HQ_{ADD}	$\frac{ADD}{Rfd}$		

Table S4. Values of RfD (mg/kg/day) for seven potentially toxic elements in the three exposure pathways

	Co	Cr	Cu	Ni	Pb	Zn	V
RfD for ingestion	0.02	0.005	0.037	0.02	0.0035	0.3	0.007
RfD for dermal absorption	0.016	0.00025	0.0019	0.001	0.00053	0.06	0.00007
RfD for inhalation	0.0000057	0.000029	0.04	0.021	0.0035	0.3	0.007

Table S5. Average non-carcinogenic exposure of potentially toxic elements in soil under three exposure pathways [mg/(kg·d)]

Elements	Adult			Child		
	ADD_{ing}	ADD_{der}	ADD_{inh}	ADD_{ing}	ADD_{der}	ADD_{inh}
Co	2.56E-05	9.64E-08	2.73E-09	1.65E-04	4.70E-07	4.55E-09
Cr	4.60E-05	5.46E-08	4.91E-09	2.96E-04	8.44E-07	8.17 E-09
Cu	2.86E-05	3.39E-06	3.04E-09	1.84E-04	5.24E-07	5.07E-09
Ni	3.26E-05	3.86E-08	6.65E-08	2.10E-04	5.97E-07	5.78E-09
Pb	6.76E-05	4.81E-07	7.21E-09	4.35E-04	1.24E-06	1.20E-08
Zn	1.68E-04	3.98E-06	1.79E-08	1.08E-03	3.07E-06	2.979E-08
V	8.10E-05	1.92E-06	1.79E-08	5.22E-04	1.49E-06	1.44E-08

Table S6. Evaluation standards of Non-carcinogenic risk

HI	Evaluation
HI ≤ 1	No significant risk of non-carcinogenic effects
1 < HI ≤ 10	Probability of the adverse health effects
HI > 10	High chronic risk

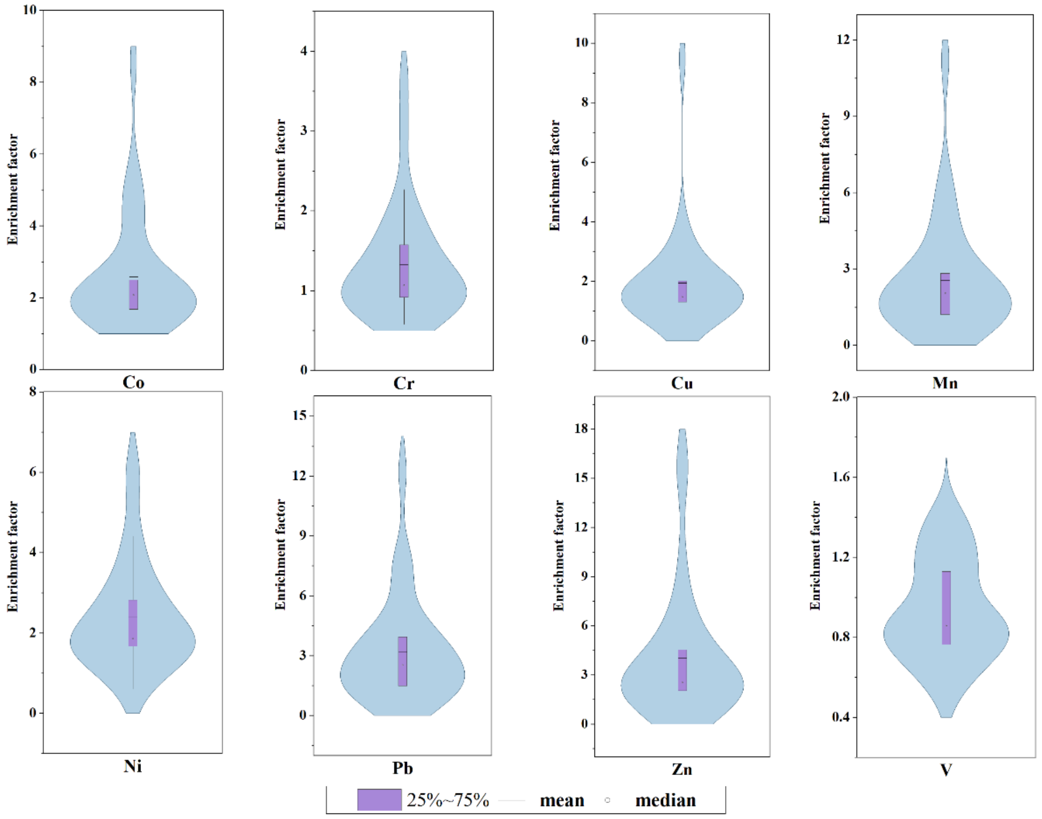

Figure S1. Enrichment factor of potentially toxic elements in soil of the study area. The solid line represents the average value of the study area

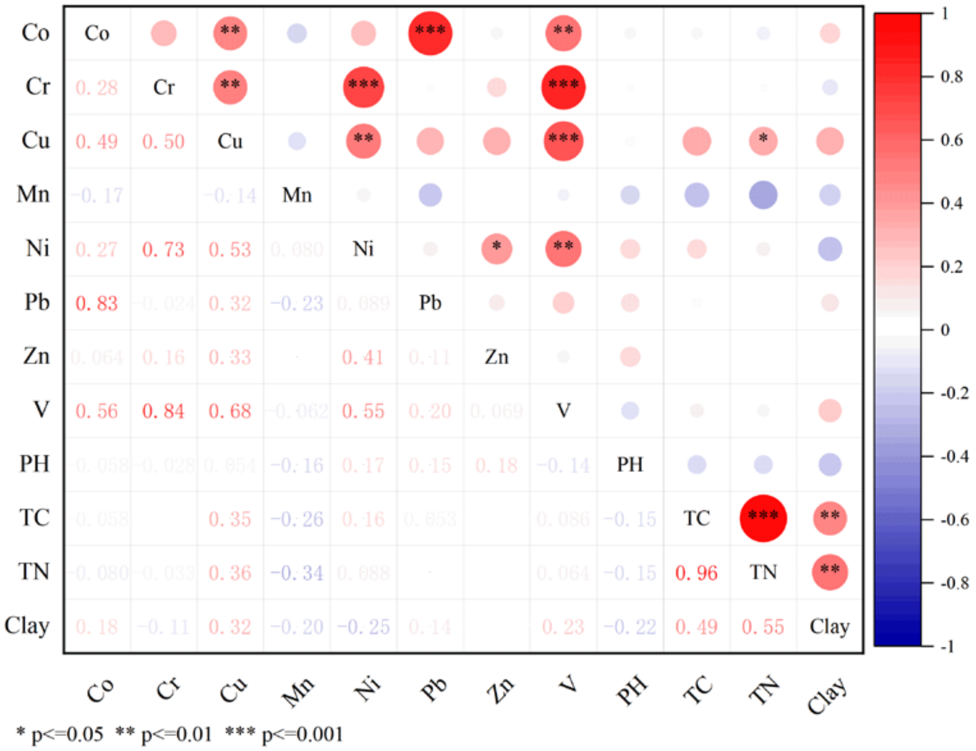

Figure S2. Correlation analysis between potentially toxic elements and physicochemical properties in soil. * represented the significant to probability level 0.05, ** represented the significant to probability level 0.01, and *** represented the significant to probability level 0.001.

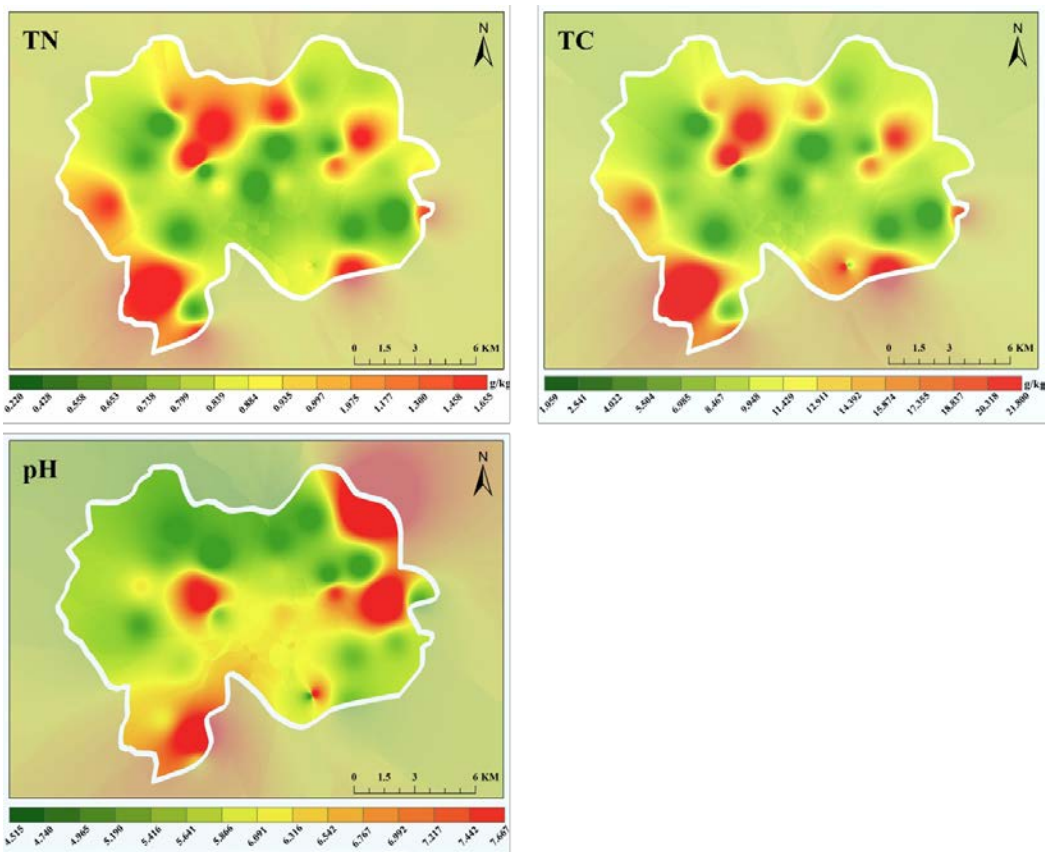

Figure S3. Distribution of physicochemical properties in soils in the study area (pH, TC, TN).

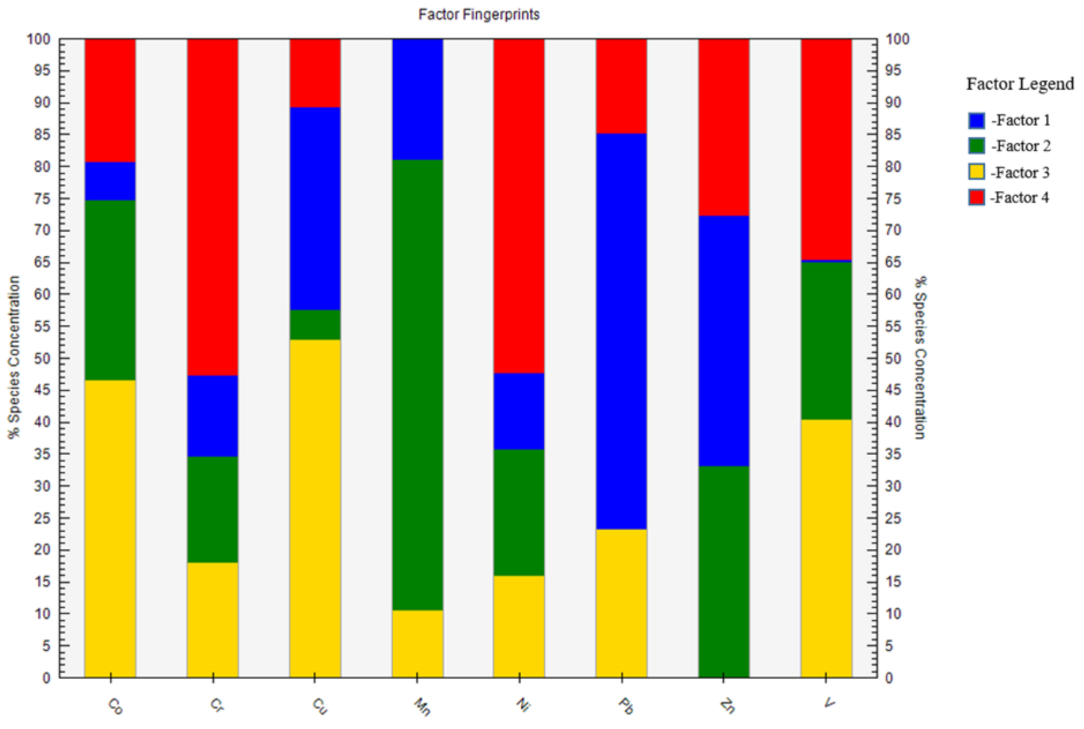

Figure S4. Contribution rate of each factor to potentially toxic elements sources

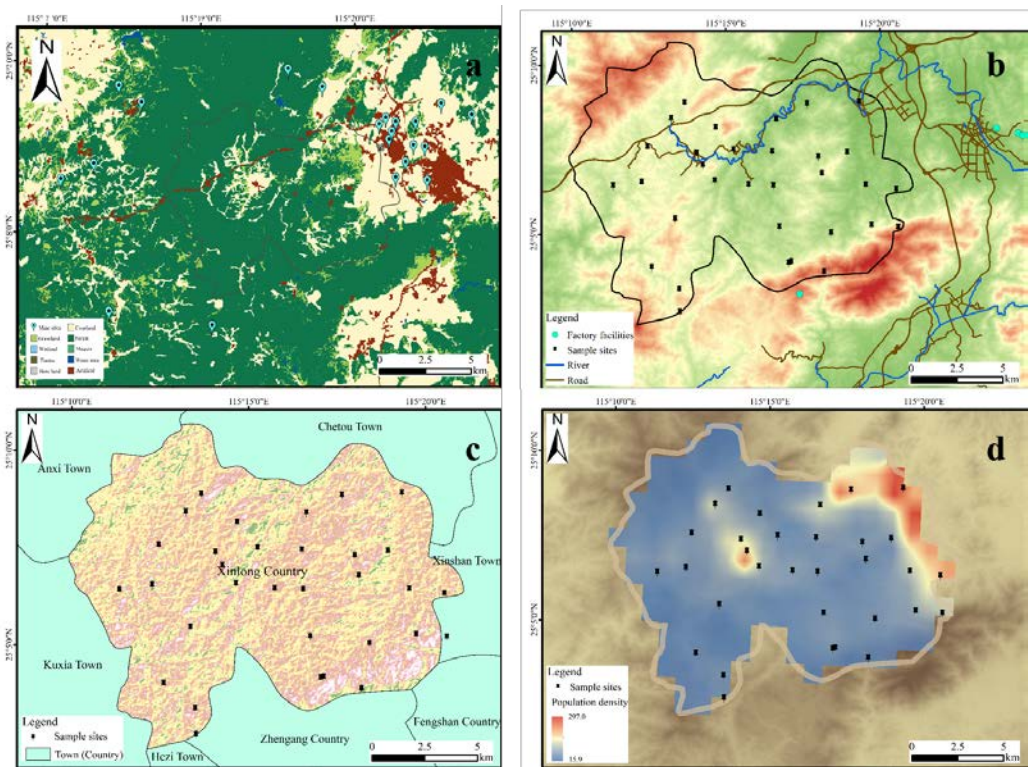

Figure S5. The thematic map of the study area. (**a**) The surface coverage of the study area and the location of the mine sites. (**b**) The network of roads and waterway and distribution of factory sites around the study area. (**c**) The subdivision of towns around the study area. (**d**) Population density in the study area and the distribution of sampling sites.
